# Hybrid nanoprobes of bismuth sulfide nanoparticles and CdSe/ZnS quantum dots for mouse computed tomography/fluorescence dual mode imaging

**DOI:** 10.1186/s12951-015-0138-9

**Published:** 2015-10-29

**Authors:** Jun Chen, Xiao-Quan Yang, Meng-Yao Qin, Xiao-Shuai Zhang, Yang Xuan, Yuan-Di Zhao

**Affiliations:** Britton Chance Center for Biomedical Photonics at Wuhan National Laboratory for Optoelectronics – Hubei Bioinformatics & Molecular Imaging Key Laboratory, Department of Biomedical Engineering, College of Life Science and Technology, Huazhong University of Science and Technology, Wuhan, 430074 People’s Republic of China; Key Laboratory of Molecular Biophysics of the Ministry of Education, College of Life Science and Technology, Huazhong University of Science and Technology, Wuhan, 430074 People’s Republic of China

**Keywords:** In vivo imaging, Bismuth sulfide, Quantum dots, CT, Fluorescence

## Abstract

**Background:**

X-ray computed tomography (CT) imaging can be used to reveal the three-dimensional structure of deep tissue with high spatial resolution. However, it cannot reveal molecular or cellular changes, and has great limitations in terms of specificity and sensitivity. Fluorescence imaging technology is one of the main methods used for the study of molecular events in vivo and has important applications in life science research. Therefore, the combination of CT and fluorescence imaging is an ideal dual-modal molecular imaging method, which can provide data on both molecular function and tissue structure, and has important research value. In a previous study, Bi_2_S_3_ nanoparticles were wrapped with quantum dots in SiO_2_ to generate CT and fluorescence imaging. However, this type of probe led to low survival and caused innegligible in vivo toxicity in mice. Therefore, it is necessary to develop new multifunctional probes that demonstrate biocompatibility and safety in vivo.

**Methods:**

A polyethylene glycol-phospholipid bilayer structure was used to synthesize hybrid clusters containing hydrophobic Bi_2_S_3_ nanoparticles and quantum dots for combined CT/fluorescence imaging. Mean particle size, polydispersity index, and zeta potential were used to study the stability over an 8-week test period. In vivo CT and fluorescence imaging experiments were performed, and the in vivo safety of the probe was evaluated, using histopathological, biochemical, and blood analyses.

**Results:**

The probe distinctly enhanced the CT contrast and had fluorescence imaging capability. In addition, the nanocomposite hybrid clusters showed a longer circulation time (>4 h) than iobitridol. The results also showed that the Bi_2_S_3_-QD@DSPE probe had good biocompatibility and safety, and did not affect normal organ functioning.

**Conclusions:**

Bi_2_S_3_-QD@DSPE hybrid clusters exhibited remarkable performance in CT angiography and fluorescence imaging in vivo.

## Background

X-ray computed tomography (CT) imaging, a traditional medical imaging technique, yields images with high spatial resolution. It can be used to determine the three-dimensional structure of deep tissue, and to show general morphological characteristics of tissues. It can also provide information on some functional and metabolic features, and has broad applications in life science research and clinical medicine [1–5]. However, it cannot reveal molecular and cellular changes characteristic of biological activity, and has great limitations in terms of specificity and sensitivity.

Fluorescence imaging technology is one of the main methods used in the study of molecular events in vivo and has important applications in life science research. Therefore, combined CT and fluorescence imaging is an ideal dual-modal molecular imaging technique [6, 7], which provides unified molecular functional and tissue structure imaging, and has important research value [8–10].

In recent years, researchers have used various metallic materials to develop CT imaging probes. Rabin et al. prepared polyvinyl pyrrolidone (PVP)-coated Bi_2_S_3_ particles which had quasi-rectangular platelet shape in water [5]. The Bi_2_S_3_-PVP particles had a high X-ray absorption efficiency and a long circulation time in vivo (>2 h). Chen et al. used a hot injection method to synthesize FePt nanoparticles (FePt NPs), which were effective for CT/MRI dual-mode imaging [11]. Hyeon et al. developed tantalum oxide nanoparticles (TaO_x_) and Rhodamine B using an in situ sol–gel reaction and used them for CT and fluorescence imaging of a rat lymph node [6]. Of these [4–11], bismuth (Bi) appears to be the most promising contrast agent, due to its large atomic number (I, 53; Ta, 73; Pt, 78; Bi, 83) and X-ray attenuation coefficient (I, 1.94; Ta, 4.30; Pt, 4.99; Bi, 5.74 cm^2^ g^−1^ at 100 keV). However, some problems remain to be solved; for example, it is difficult to control the size of Bi_2_S_3_ nanoparticles, and their surface is also difficult to modify, thus hindering their application as CT-based multifunctional probes. It is for this reason that use of a Bi contrast agent with an optical probe has seldom been reported. However, a variety of fluorescent materials have been used in fluorescence imaging, for example, fluorescent dyes and quantum dots (QDs). Compared with fluorescent dyes, which have obvious disadvantages in direct biological imaging, QDs show higher brightness and photo-stability, broad excitation profiles, narrow/symmetric emission spectra, and high quantum yields, and are therefore suited for use in many areas of biomedical imaging research.

**Fig. 1:**
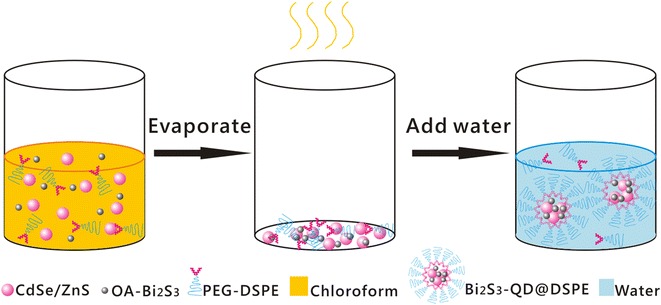
Schematic illustration of the procedures used for synthesis of Bi_2_S_3_-QD@DSPE hybrid nanoprobes

In a previous study [12], we wrapped Bi_2_S_3_ nanoparticles with quantum dots in SiO_2_. These composite nanoparticles showed promise for use in CT and fluorescence imaging in vitro and in vivo; however, they also showed in vivo toxicity for mice and led to a low survival rate. 1,2-Dipalmitoyl-*sn*-glycero-3-phosphoethanolamine-N-[methoxy(polyethylene glycol)-2000] (PEG-DSPE) has been used by some groups to prepare in vivo probes. For example, Sailor et al. used PEG-phospholipid to prepare hybrid nanoparticles that contained magnetic iron oxide nanoparticles, QDs, and the anticancer drug doxorubicin for simultaneous targeted drug delivery and dual-mode near-infrared fluorescence imaging and MRI of diseased tissue in vitro and in vivo [13]. Lu et al. used PEG 2000-DSPE to coat Yb-based nanoparticles as a high-performance CT contrast agent for in vivo angiography and bimodal image-guided lymph node mapping, and they found that the gradual enhancement of signals for the liver and spleen continued for >2 h [14]. In the present study, PEG 2000-DSPE was used to synthesize hybrid clusters containing hydrophobic Bi_2_S_3_ nanoparticles and quantum dots for combined CT/fluorescence imaging (Fig. 1). For CT imaging of the liver and spleen, the particles produced a clear contrast enhancement after 30 min, and the enhancement continued for >4 h; for fluorescence imaging, the liver and kidneys continued to show fluorescence after 24 h, indicating that the probes have a long circulation time. The results also showed that the Bi_2_S_3_-QD@DSPE probes show good biocompatibility and safety, and did not affect normal organ functioning. Thus, our results show that Bi_2_S_3_-QD@DSPE hybrid clusters exhibit remarkable performance in CT angiography and fluorescence imaging in vivo.

## Results and discussion

**Fig. 2:**
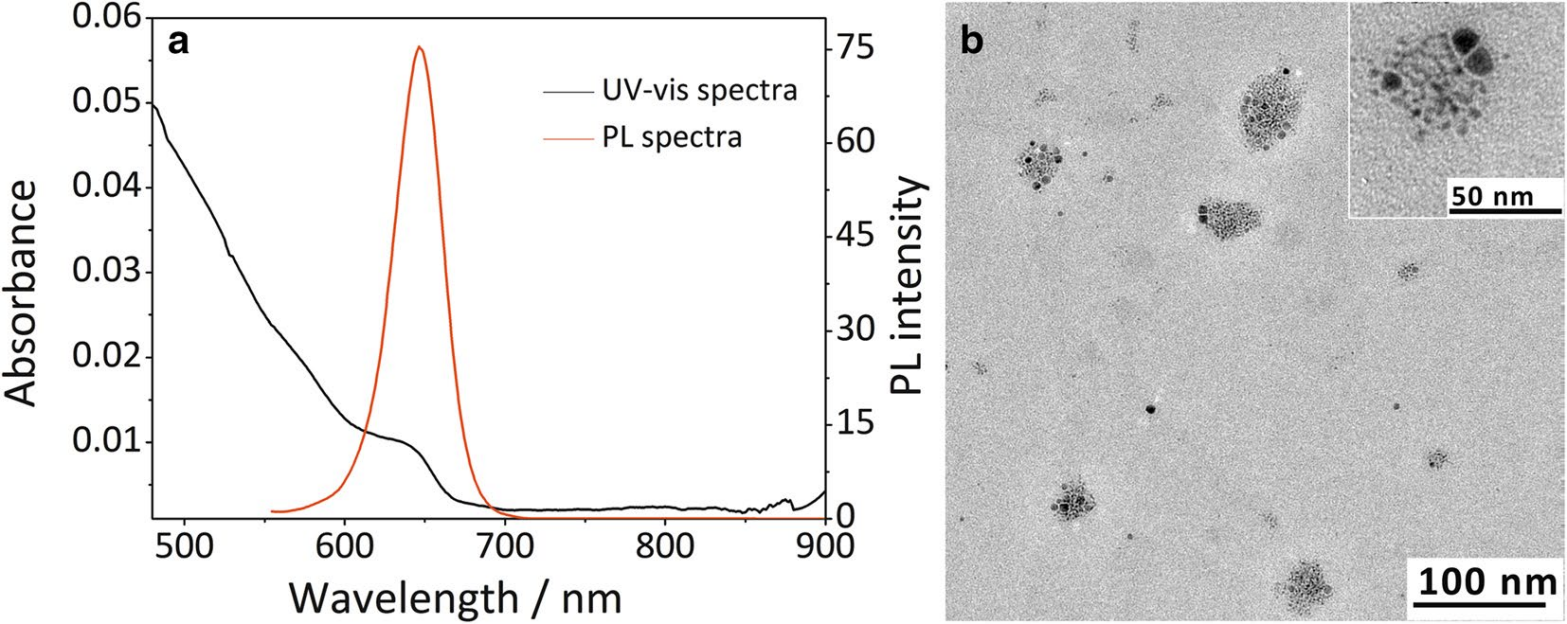
UV-vis absorption spectra and fluorescence spectra of QD (**a**) and TEM image of Bi_2_S_3_-QD@DSPE hybrid clusters (**b**)

**Fig. 3:**
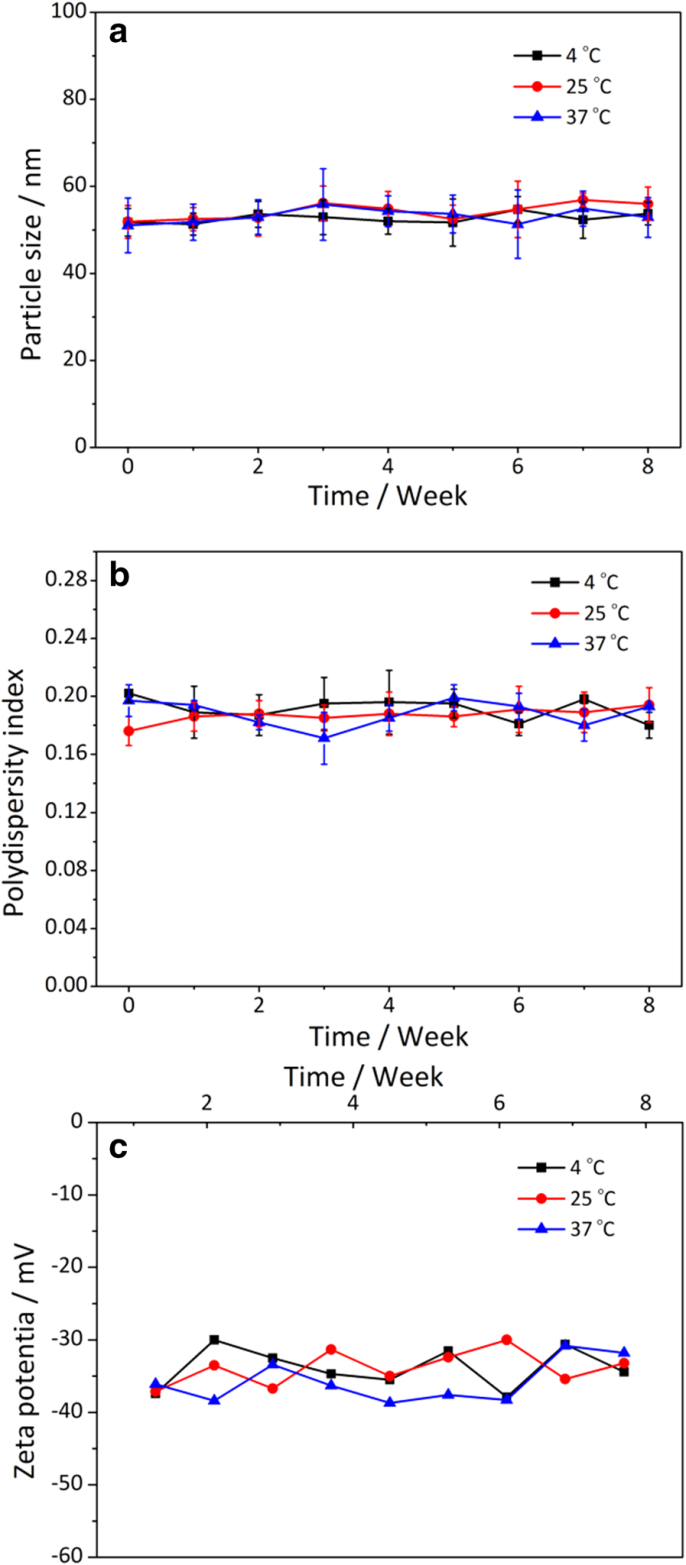
Physical stability of Bi_2_S_3_-QD@DSPE during storage at 4, 25, and 37 °C. Changes in mean particle size (**a**), polydispersity index (**b**) and zeta potential (**c**) of Bi_2_S_3_-QD@DSPE were examined

To synthesize Bi_2_S_3_-QD@DSPE, we first prepared hydrophobic Bi_2_S_3_ nanoparticles and QDs. The photoluminescence (PL) spectra of the QDs showed that they had an emission wavelength of approximately 650 nm (Fig. 2a) with a full-width-at-half-maximum (FWHM) of 28 nm. TEM images showed that these hybrid clusters were 30–40 nm in diameter (Fig. 2b), which was in good agreement with the average diameters determined by dynamic light scattering measurements (Fig. 3a). After the synthesis of Bi_2_S_3_-QD@DSPE, the stability of hybrid clusters was evaluated by examining changes in the mean particle size, polydispersity index (PDI), and zeta potential during storage at 4, 25, and 37 °C. Figure 3 illustrates that the mean size, PDI, and zeta potential of the probes were approximately 50 nm, 0.188, and −35.7 mV, respectively, and the probes did not show any significant changes in particle size, PDI, or zeta potential when maintained at 4, 25, and 37 °C for the 8-week test period, indicating that the probes show sufficient stability. The probes showed a negative zeta potential due to dissociation of the phosphate group in the PEG-phospholipid moiety, consistent with previous reports [15, 16].

**Fig. 4:**
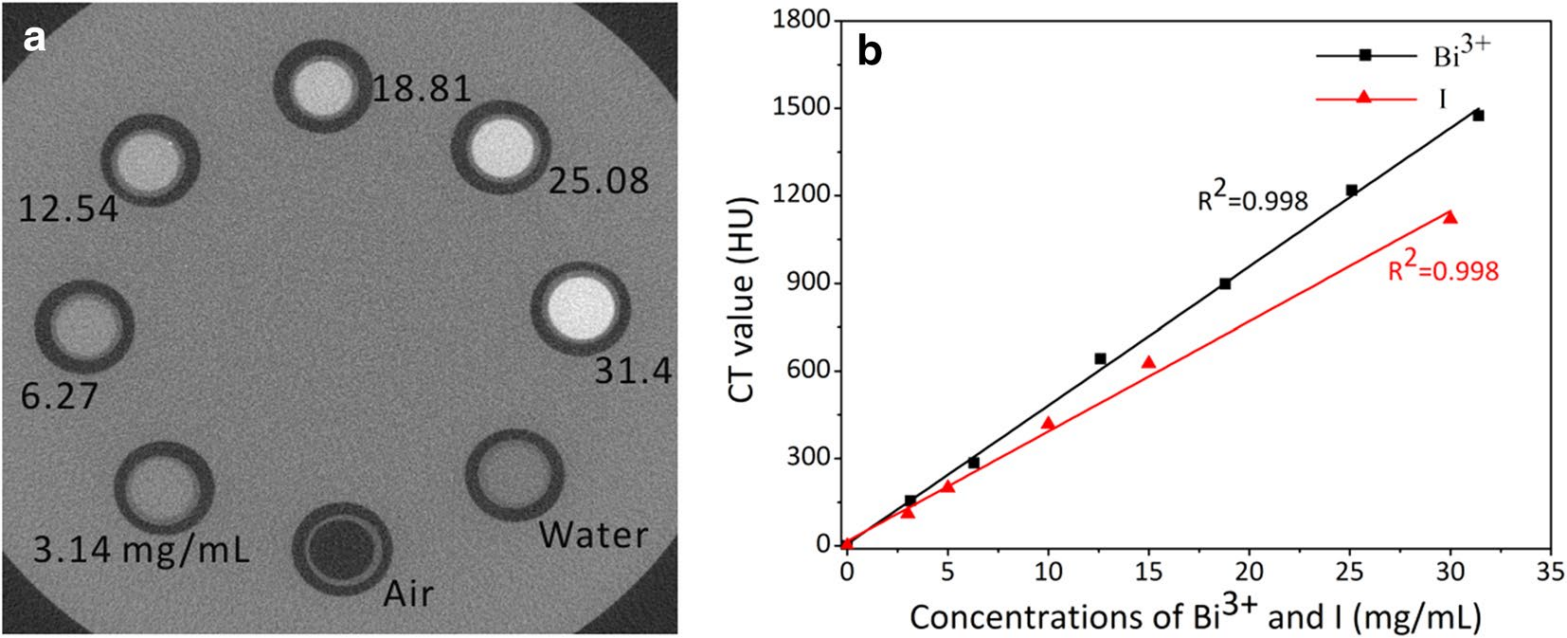
Phantom images of Bi_2_S_3_-QD@DSPE hybrid clusters as a function of concentration, using X-ray CT imaging (**a**) and HU measurements (**b**)

Various concentrations of Bi_2_S_3_-QD@DSPE were dispersed in deionized water. As the concentration of Bi_2_S_3_-QD@DSPE increased, the Hounsfield unit (HU) values obtained using the in-house-built CT system increased linearly (Fig. 4a, b). The probes showed higher HU values and CT contrast effects than the same concentration of iopromide.

**Fig. 5:**
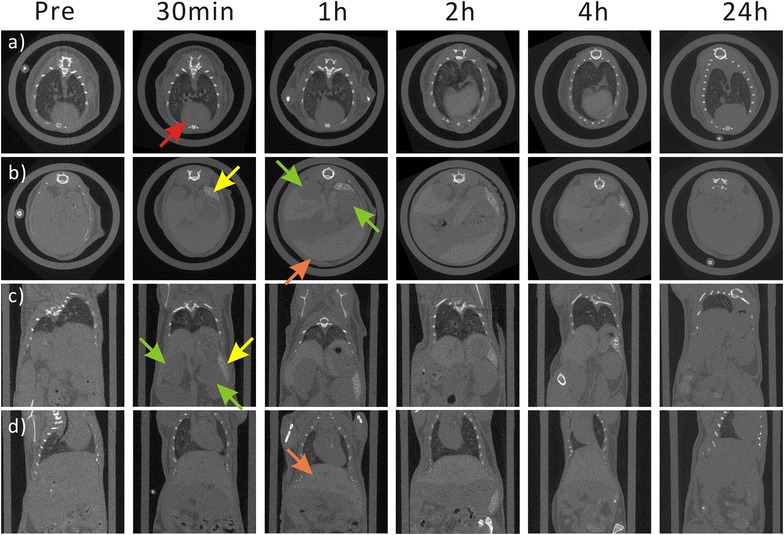
In vivo X-ray CT imaging. Serial CT sections (**a**, **b**) and coronal views (**c**, **d**) of a mouse at different time points after injection of Bi_2_S_3_-QD@DSPE hybrid clusters in solution (0.6 mg Bi/g body weight) into the tail vein [heart (**a**, *red arrow*), liver (*orange arrow*), spleen (*yellow arrow*), and kidney (**b**, *green arrow*)]

To study their biodistribution, Bi_2_S_3_-QD@DSPE particles were injected into the tail veins of mice. Distribution of the probes was tracked by X-ray CT imaging before injection and at 30 min, 1, 2, 4, and 24 h after injection. Figure 5a shows that CT contrast of the heart (red arrow) was enhanced after 30 min and decreased 1 h after administration of Bi_2_S_3_-QD@DSPE, likely because the probes spread to the heart and then to other organs with blood circulation. Serial CT sectional and coronal views (Fig. 5b–d) revealed that the liver (orange arrow) and spleen (yellow arrow) showed clear contrast enhancement after 30 min, and that the enhancement continued for >4 h, indicating that the probes show a long circulation time. However, contrast was weak after 24 h, which may imply that most of the probes were eventually metabolized and did not accumulate in vivo. Furthermore, the HU values of organs at several time points were tested (Table 1). After injection, the HU value of the heart began to peak at approximately 30 min, and spleen and liver signal intensity increased until they peaked at 1–2 h (spleen: 107.9–513.4; liver: 123.1–257.1). The contrast effect in all of the examined organs returned to normal levels after 24 h, as shown in the serial CT images.

**Table 1:** CT values of the heart, kidney, liver and spleen of mouse before the injection (previous) and at indicated time intervals after the injection

	Previous	30 min	1 h	2 h	4 h	24 h
Heart	81.8	196.2	123.1	81.8	91.9	102.1
Kidney	113.0	170.9	111.5	107.2	144.1	114.4
Liver	123.1	192.6	250.5	257.1	256.3	131.8
Spleen	107.9	474.3	513.4	488.1	393.9	131.1

**Fig. 6:**
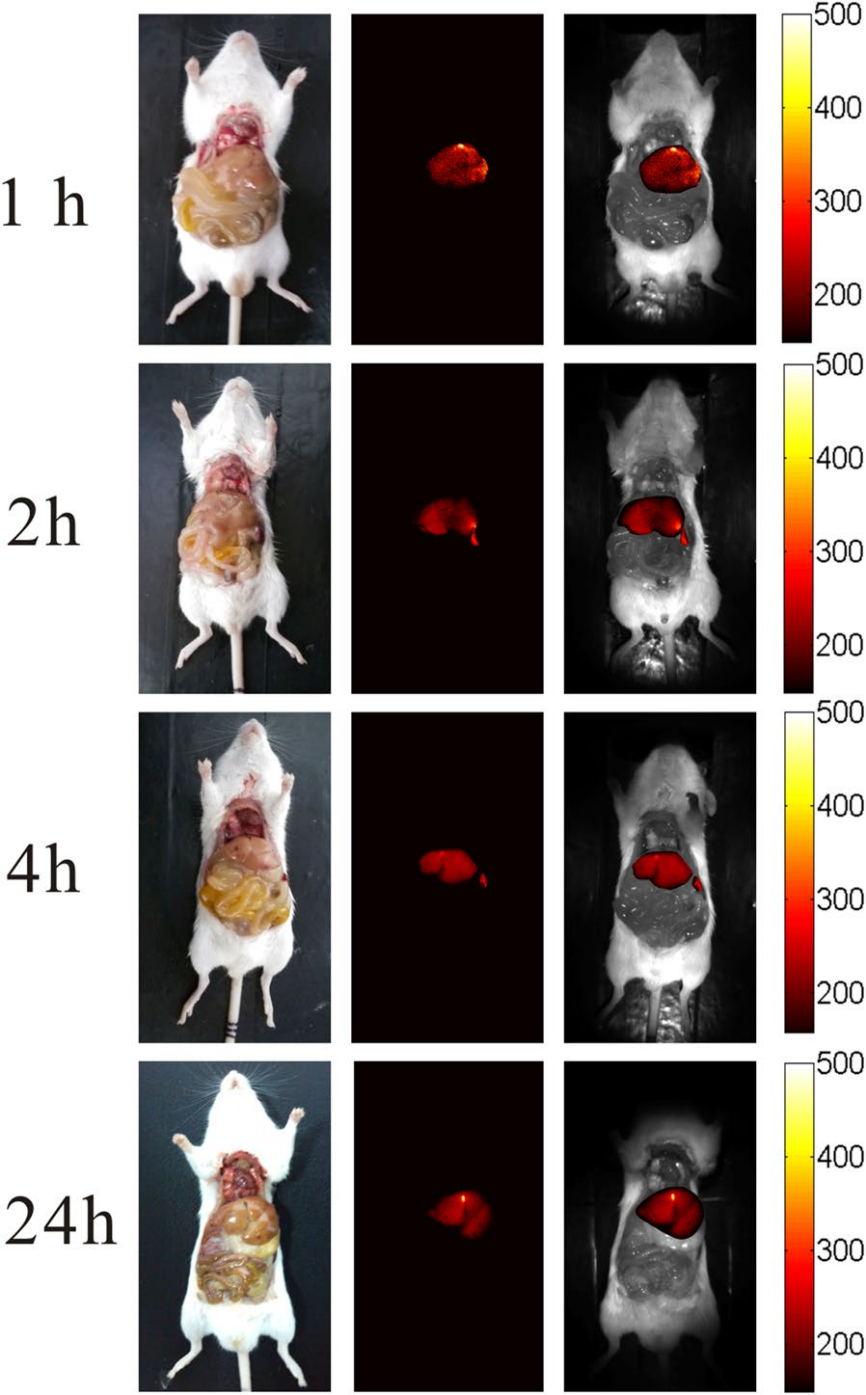
In vivo whole-body optical imaging after injection with Bi_2_S_3_-QD@DSPE hybrid clusters in solution [bright-field (*left*), fluorescent (*middle*), and merged (*right*) images]

**Fig. 7:**
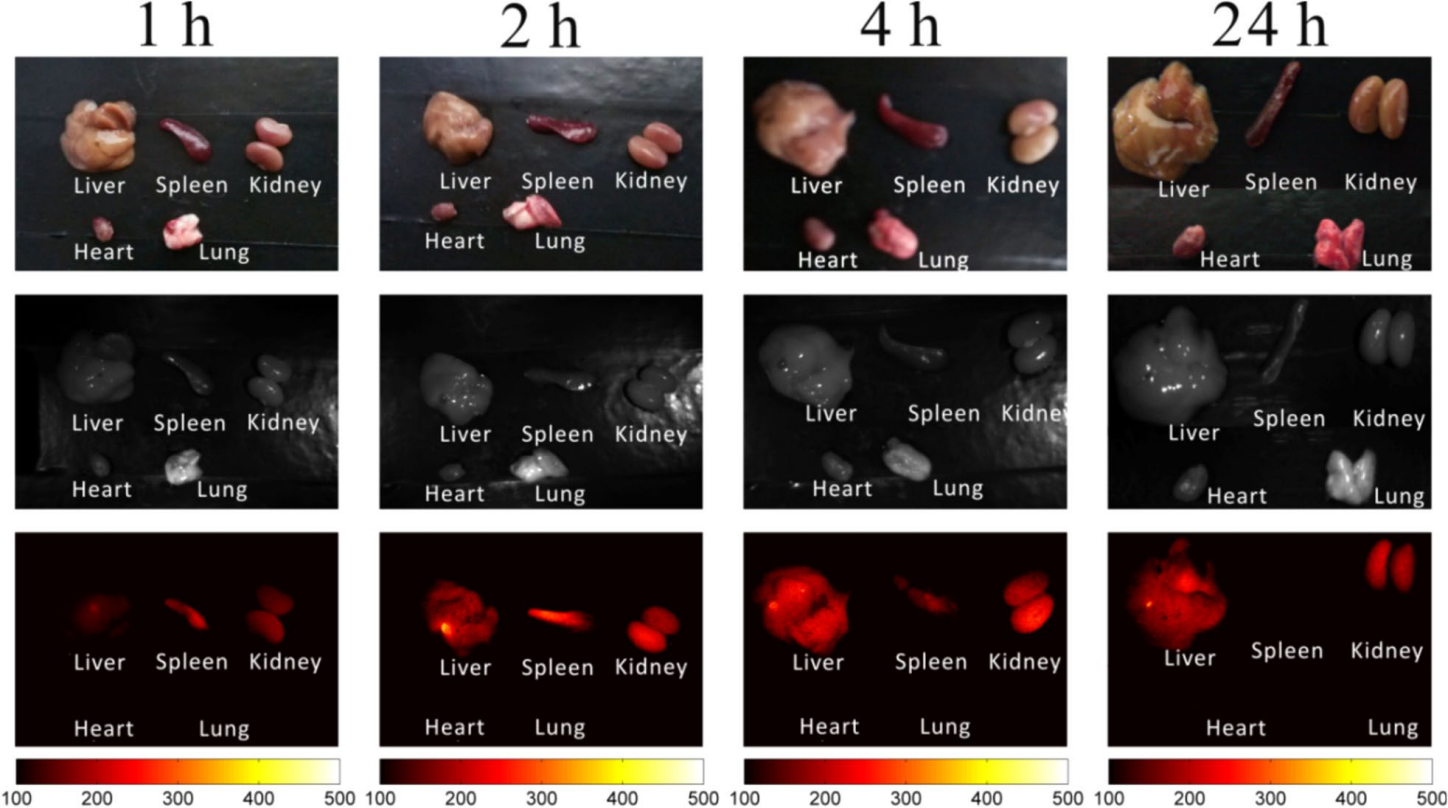
In vivo optical imaging of organs after injection with Bi_2_S_3_-QD@DSPE hybrid clusters in solution at 1, 2, 4, and 24 h (bright-field (*upper*, *color image*), bright-field (*middle*, *black-and-white* *image*), and fluorescent (*lower*) images)

The excitation wavelength of our in house-built imaging system for Bi_2_S_3_-QD@DSPE was 500 nm and showed limited optical imaging capability in vivo, and therefore the mice were sacrificed after CT imaging. In this study, the auto-fluorescence signal has been deducted from all of the results for fluorescence imaging. Distribution of the probes was tracked by fluorescence imaging at 1, 2, 4, and 24 h after injection. Figures 6 and 7 show that probes were enriched in the liver, spleen, and kidneys at 1 h after injection; the enrichment lasted for 4 h, in agreement with the results of CT imaging. In the spleen, fluorescence intensity began to peak at 1–2 h, which may imply that a large number of macrophages initiated phagocytosis after the injection, that most probes were metabolized at approximately 2 h, and had not accumulated in the spleen 24 h after injection; this may explain why no spleen uptake was observed at 24 h post-injection in the optical images. Interestingly, fluorescence imaging of the kidney and liver disagreed with CT imaging of these organs at 24 h, possibly because the sensitivity of the CT imaging system is lower than that of the fluorescence imaging system, and it was difficult to detect the small amount of probes remaining. These results indicate that hybrid Bi_2_S_3_-QD@DSPE nanoprobes have both CT and fluorescence imaging capability in vivo.

**Fig. 8:**
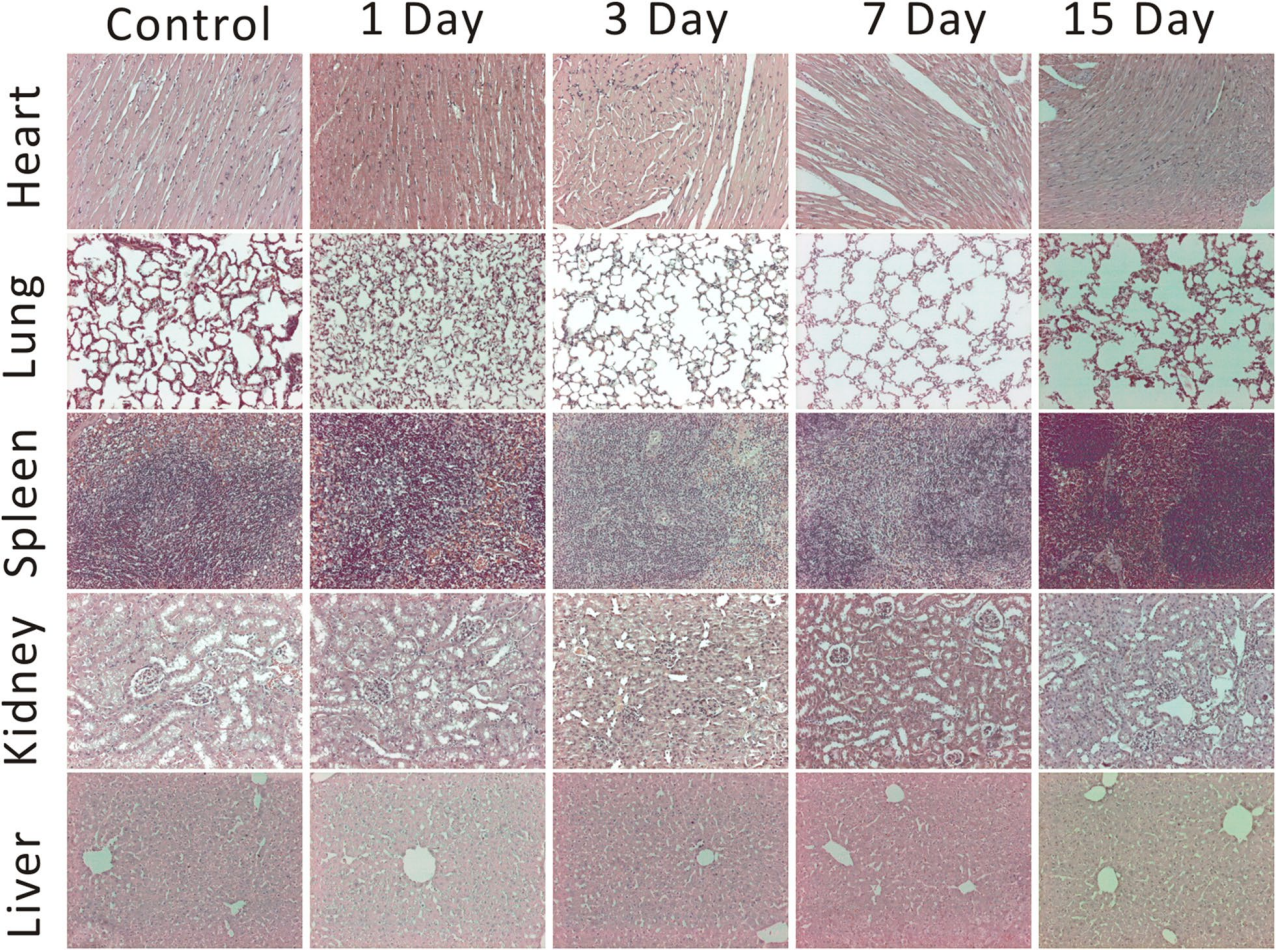
H&E-stained tissue sections from mice to observe the time course of the histological changes in the heart, lung, spleen, kidney, and liver tissue of mice receiving a single intravenous injection of PBS (control) or Bi_2_S_3_-QD@DSPE hybrid clusters solution (0.6 mg Bi/g body weight)

**Fig. 9:**
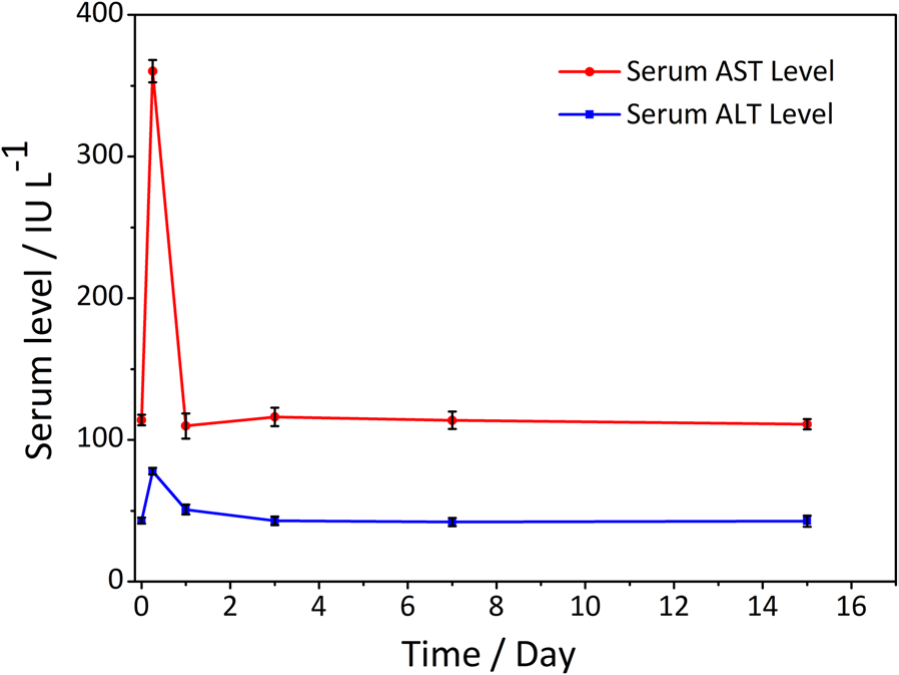
Changes in serum liver enzyme levels as a function of time (n = 5)

To determine whether Bi_2_S_3_-QD@DSPE caused any harmful effects or disease, the long-term toxicity of the probes was investigated by monitoring histological changes in several susceptible organs including the heart, lung, spleen, kidney, and liver for more than 2 weeks. Mice were dissected at 1, 3, 7, and 15 days after the injection of a single dose (0.6 mg Bi/g body weight) of Bi_2_S_3_-QD@DSPE. Hematoxylin and eosin (H&E) staining of organs showed no significant evidence of tissue damage or adverse effects of the nanoparticles on the organs (Fig. 8). We also measured serum levels of alanine aminotransferase (ALT) and aspartate aminotransferase (AST) over time to determine the effect on liver function. Figure 9 showed that the ALT and AST levels increased transiently after the injection and declined rapidly until they returned to normal. Jain et al. used magnetic nanoparticles in rats and showed similar results [17]. Thus, our probes exhibit little toxicity in the liver. Given that blood can reflect the condition of tissues and organs, we performed blood analysis on mice that received either a single intravenous injection of 150 µL phosphate buffered saline (PBS) (control) or Bi_2_S_3_-QD@DSPE (0.6 mg/g) at the indicated time. Neither group showed significant differences in any of the following four indexes: white blood cell count (WBC), red blood cell count (RBC), hemoglobin (HGB), or platelet count (PLT), and all indexes were in the reference range (Table 2) [18]. These results further confirmed that Bi_2_S_3_-QD@DSPE nanoprobes had no significant effect on organs, tissues, or blood, and showed remarkable biocompatibility and promise as a contrast agent.

**Table 2:** Blood analysis of mouse that received single intravenous injection of 150 µL PBS (control) or Bi_2_S_3_-QD@DSPE (0.6 mg/g dose in PBS) followed by dissection at the indicated times (n = 5)

		WBC^a^,10^9^/L	RBC^b^,10^12^/L	HGB^c^,g/L	PLT^d^,10^9^/L
Reference values	6 weeks	7.25–10.13	10.04–11.09	155.57–168.70	992.31–1181.23
	8 weeks	8.08–11.50	10.13–13.19	142.47–188.33	734.60–1270.30
1 day	PBS	9.52 ± 0.74	10.62 ± 0.20	161.20 ± 2.86	998.00 ± 12.59
	Probe	9.78 ± 0.36	10.70 ± 0.17	160.60 ± 6.80	1023.00 ± 50.98
3 day	PBS	9.80 ± 0.45	10.88 ± 0.18	164.60 ± 2.70	1022.40 ± 41.11
	Probe	9.84 ± 0.44	10.75 ± 0.23	162.40 ± 7.33	1012.60 ± 33.09
7 day	PBS	9.72 ± 0.33	10.72 ± 0.38	165.81 ± 1.92	1033.40 ± 34.09
	Probe	9.78 ± 0.26	10.78 ± 0.36	163.00 ± 5.34	998.60 ± 36.67
15 day	PBS	10.72 ± 0.15	11.84 ± 0.34	172.20 ± 7.95	970.60 ± 68.72
	Probe	10.46 ± 0.15	11.92 ± 0.63	176.20 ± 6.10	1015.40 ± 39.17

^a^white blood cell count

^b^red blood cell count

^c^hemoglobin

^d^platelet count

## Conclusion

In summary, composite core–shell nanoparticles containing Bi_2_S_3_ nanoparticles and QDs within a single PEG-modified phospholipid were prepared. These nanoprobes exhibit effective dual-mode imaging in vivo, and have the advantages of long circulation time and low toxicity. However, because the excitation wavelength of our in house-built imaging system for the contrast agent probe is 500 nm, which is not conducive to noninvasive optical imaging in vivo, we intend to develop a multi-mode probe that can fully exploit the advantages conferred by the near-infrared range of QDs, in future work.

## Methods

### Materials

Bismuth neodecanote, oleic acid (90 %), cadmium oxide (99.99 %), bis(trimethylsilyl) sulfide ((TMS)_2_S), hexadecylamine (90 %), tri-n-octylphosphine oxide (90 %), and trioctylphosphine (97 %) were purchased from Aldrich. Oleyl amine (90 %) was purchased from Aladdin. Thioacetamide and selenium powder were purchased from Sinopharm. 1,2-Dipalmitoyl-sn-glycero-3-phosphoethanol-amine-N-[methoxy(polyethylene glycol)-2000] (PEG-DSPE, 99 %) was purchased from Avanti. All chemicals were used as received, unless otherwise stated.

### Characterization

Nanoparticle spectra were measured using a 2550 UV–visible spectrophotometer (Japan) and a LS-55 spectrophotometer (USA). Elemental analysis of the resultant hybrid clusters was performed using an inductively coupled plasma atomic emission spectrometer (Germany). Animal blood analysis was carried out on a SP-4430 dry biochemical analyzer (Japan) and a CA-700 automatic blood analyzer (China). TEM images were captured using a Tecnai G20 U-Twin transmission electron microscope (USA).

### Synthesis of Bi_2_S_3_-QD@DSPE

Based on previously reported methods, we prepared Bi_2_S_3_ nanoparticles coated with oleic acid [4]. Core–shell CdSe/ZnS quantum dots with an emission wavelength of 650 nm were synthesized using a previously described two-step method [19, 20]. After addition of PEG-DSPE, hydrophobic Bi_2_S_3_ and QD were modified and transformed into hydrophilic nanocomposite hybrid clusters, Bi_2_S_3_-QD@DSPE. The main steps were performed according to previously reported methods [13, 21]. In brief, Bi_2_S_3_ nanoparticles and QD were mixed with PEG-DSPE in chloroform. Bi_2_S_3_-QD@DSPE hybrid clusters were prepared by evaporating the organic solvent in a rotary evaporator under vacuum with 300r/min rotation speed for 30 min and then flushing with a N_2_ stream to remove any residual traces of organic solvent. The lipid film, deposited in the reaction vial, was hydrated with water and subjected to ultrasonication. The resulting dispersion was purified by centrifugation using a Millipore filter (Centrifugal Filter Devices, 100 K) to remove excess PEG-DSPE. The final nanoparticles were redispersed in PBS for use in further experiments.

### CT imaging of Bi_2_S_3_-QD@DSPE

Various concentrations of Bi_2_S_3_-QD@DSPE (3.14, 6.27, 12.54, 18.81, 25.08, and 31.4 mg of Bi/mL) dispersed in deionized water were prepared in 1.5-mL microtubes. A high-resolution CT system was constructed and applied for imaging, using the following imaging parameters: spatial resolution of 100 µm, tube voltage of 50 kV, and tube current of 0.800 mA.

### Measurement, polydispersity index (PDI), and zeta potential of Bi_2_S_3_-QD@DSPE

The mean particle size, PDI, and zeta potential of Bi_2_S_3_-QD@DSPE were measured during storage at 4, 25, and 37 °C using a nano-ZS90 dynamic light scattering (Malvern, UK) according to the manufacturer’s instructions.

### In vivo CT and fluorescence imaging

Balb/C mice with an average age of 6–8 weeks were purchased from the Hubei Medical Laboratory Animal Center. All animal studies were approved by the Animal Experimentation Ethics Committee of Huazhong University of Science and Technology. Balb/C mice (male) was anesthetized using 2 % chloral hydrate and 10 % urethane. Subsequently, Bi_2_S_3_-QD@DSPE (0.6 mg Bi/g body weight) was injected through the tail vein into the mice and CT imaging was performed at appropriate time points (pre-injection, 30 min, 1, 2, 4, and 24 h) after tail vein injection. The mice were then perfused and cleared with PBS for fluorescence imaging. Experiments were performed in quintuplicate using 5 mice. A high-resolution CT system was constructed and applied for imaging, using the following imaging parameters: spatial resolution of 100 µm, tube voltage of 50 kV, and tube current of 0.800 mA. Fluorescence images of Bi_2_S_3_-QD@DSPE were acquired using a filter set (excitation filter: 469/35 nm; emission filter: 655/40 nm) and calibrated using an auto-fluorescent background filter set (excitation filter: 396/40 nm; emission filter: 655/40 nm). The CT and fluorescence imaging systems were both built in-house [22].

### In vivo safety evaluation of probes

The male Balb/C mice were randomly assigned to two groups, with five in each group. After receiving a single intravenous injection of either PBS (control) or probes, 250-µL blood samples were collected from each mouse by extraction of orbital venous plexus blood, including 200 µL of blood for liver function tests (ALT and AST assays, carried out on a SP-4430 dry biochemical analyzer) and 50 µL of blood for blood analysis (measured using a CA-700 automatic blood analyzer). Organs, including heart, lung, spleen, kidney, and liver were stained with H&E at the appropriate time points (1, 3, 7, and 15 d) and observed under a light microscope at 10× magnification.

## Abbreviations

CT: computed tomography
QDs: quantum dots
PEG-DSPE: 1,2-dipalmitoyl-sn-glycero-3-phosphoethanolamine-N-[methoxy (polyethylene glycol)-2000]
PDI: polydispersity index
H&E: hematoxylin and eosin
ALT: alanine aminotransferase
AST: aspartate aminotransferase
WBC: white blood cell count
RBC: red blood cell count
HGB: hemoglobin
PLT: platelet count
PBS: phosphate buffered saline
